# Differentially methylated CpG island within human XIST mediates alternative P2 transcription and YY1 binding

**DOI:** 10.1186/s12863-014-0089-4

**Published:** 2014-09-09

**Authors:** Andrew G Chapman, Allison M Cotton, Angela D Kelsey, Carolyn J Brown

**Affiliations:** 1 Department of Medical Genetics, Molecular Epigenetics Group, Life Sciences Center, 2350 Health Sciences Mall, Vancouver V6T 1Z3, BC, Canada

**Keywords:** XIST, X-chromosome inactivation, Alternative promoter, YY1, DNA methylation, Long non-coding RNA, RNA FISH, DNase hypersensitivity site

## Abstract

**Background:**

X-chromosome inactivation silences one X chromosome in females to achieve dosage compensation with the single X chromosome in males. While most genes are silenced on the inactive X chromosome, the gene for the long non-coding RNA XIST is silenced on the active X chromosome and expressed from the inactive X chromosome with which the XIST RNA associates, triggering silencing of the chromosome. In mouse, an alternative *Xist* promoter, P2 is also the site of YY1 binding, which has been shown to serve as a tether between the Xist RNA and the DNA of the chromosome. In humans there are many differences from the initial events of mouse *Xist* activation, including absence of a functional antisense regulator *Tsix*, and absence of strictly paternal inactivation in extraembryonic tissues, prompting us to examine regulatory regions for the human *XIST* gene.

**Results:**

We demonstrate that the female-specific DNase hypersensitivity site within *XIST* is specific to the inactive X chromosome and correlates with transcription from an internal P2 promoter. P2 is located within a CpG island that is differentially methylated between males and females and overlaps conserved YY1 binding sites that are only bound on the inactive X chromosome where the sites are unmethylated. However, YY1 binding is insufficient to drive P2 expression or establish the DHS, which may require a development-specific factor. Furthermore, reduction of YY1 reduces *XIST* transcription in addition to causing delocalization of *XIST*.

**Conclusions:**

The differentially methylated DNase hypersensitive site within *XIST* marks the location of an alternative promoter, P2, that generates a transcript of unknown function as it lacks the A repeats that are critical for silencing. In addition, this region binds YY1 on the unmethylated inactive X chromosome, and depletion of YY1 untethers the XIST RNA as well as decreasing transcription of XIST.

## 1 Background

X-chromosome inactivation (XCI) is a process in mammalian females allowing for dosage compensation of X-linked genes between the sexes [1]. XCI is controlled by an alternatively spliced, long non-coding RNA called *XIST/Xist*[2],[3] that is up-regulated at the onset of XCI and is necessary for silencing [4]-[6]*. XIST/Xist* coats the future inactive X chromosome (Xi) [7] and recruits chromatin modifications that enable transcriptional silencing of the majority of genes on the Xi. Therefore, to achieve XCI, a female cell must ensure monoallelic up-regulation of *XIST/Xist* while a male cell must fully repress *XIST/Xist* expression*.*

*XIST/Xist* regulation is dependent on promoter activity but very little is known about the mechanisms controlling *XIST/Xist* promoter action beyond the minimal promoter sequences required for transcription [8],[9]. An alternative promoter for mouse *Xist* called P2 has been observed and is located ~1500 bp downstream of the canonical promoter (P1) [10]. In humans, a region homologous to P2, in combination with P1, showed higher expression of a reporter gene relative to clones containing P1 alone, providing indirect evidence for conservation of P2 activity [11]. In both mice and humans this region is contained within a CpG island that becomes methylated on the Xa to maintain *XIST* repression [12]. Demethylation of this island is correlated with reactivation of *XIST* in human [13] and mouse [14] somatic cells. Moreover, the transcription factor YY1 has recently been shown to be central to the *cis* specificity of *Xist* by tethering Xist RNA to the *Xist* genomic locus at binding sites located within the *Xist* CpG island [15].

Beyond the *XIST/Xist* promoter, the minimal region required for XCI, the X-inactivation centre, (*XIC*) [16],[17], is believed to contain the regulatory elements essential for proper regulation of *XIST/Xist.* An invaluable tool in understanding *Xist* regulation has been mouse embryonic stem (ES) cells due to their ability to undergo XCI during cellular differentiation [18]. Studies in mouse ES cells have revealed a network of activating or repressing factors that are either contained within the *Xic* or act through the *Xic*. Importantly, a non-coding RNA antisense to *Xist,* called *Tsix*, has been proven to be a crucial repressor of *Xist* during cellular differentiation, blocking *Xist* on the Xa and becoming downregulated on the Xi to allow *Xist* expression [19],[20]. Several other regulatory factors also act at the *XIC*, including CTCF, which represses *Xist* upregulation [21],[22], and RNF12 [23] and the non-coding RNAs Jpx [22],[24] and Ftx [25], which activate *Xist* expression.

In humans, ES cells show considerable variation between lines and culture conditions in terms of their XCI status [26]-[28]. The variability in human ES cells has hindered studies of *XIST* regulation but several lines of evidence suggest that human and mouse *XIST/Xist* regulation may be different. The initial XCI in mouse is paternally imprinted, with extraembryonic tissues maintaining inactivation of the paternal X. In humans, XCI is random in all tissues with respect to the X chromosome that undergoes inactivation, and the timing of the initiation and role of any parental imprint has not been resolved ([29] and reviewed in [30]). Thus the extent to which the molecular machinery detailed for the initial choice of X to inactivate in mice is functional in other species remains to be determined. *TSIX/Tsix* has undergone substantial divergence between species [31],[32]. In the limited human cells reported to transcribe *TSIX*, the antisense RNA is not capable of repressing *XIST* nor does transcription extend across the *XIST* promoter [32], a property crucial to *Tsix* repression of *Xist* in mouse [33], and fluorescent *in situ* hybridization (FISH) of *TSIX* transcripts indicates transcription is originating from the Xi rather than the Xa [34]. In addition to differences in *TSIX/Tsix,* the entire *XIC/Xic* is poorly conserved, suggesting that *cis*-regulatory elements between species may be rapidly evolving [30],[31], and *Xist/XIST* appears to be specific to eutheria with a different long non-coding RNA, Rsx, sharing similar properties in marsupials [35].

DNase I hypersensitivity (DHS) is commonly used to identify regulatory elements due to their open chromatin structure being sensitive to digestion by DNase I. Within the *XIC*, DHS mapping has revealed three putative regulatory elements, one downstream of *XIST* and two upstream of *XIST*, that do not appear to be conserved between humans and mice [36]. In this paper we investigate a strong DHS site located within the human *XIST* CpG island and find that hypersensitivity correlates with P2 promoter usage and is independent of YY1 binding.

## 2 Results

### 2.1 DNase Hypersensitivity Site (DHS) mapping

**Figure 1:**
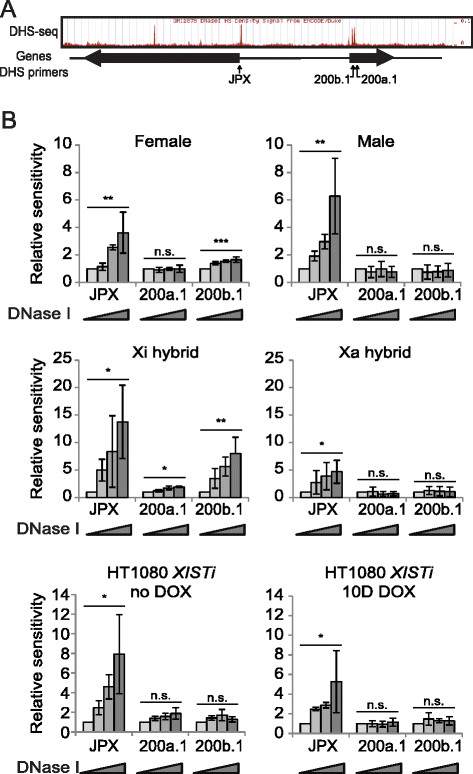
**Xi-specific DNase I hypersensitive site ~1.5 kb within*XIST.*A)** Schematic indicating DHS sites from ENCODE genome-wide survey with locations of primers used for qPCR-based DHS assay. Shading of bars reflects increasing amounts (0, 10, 20 or 40 U) of DNase. **B)** qPCR-based DHS assay on female and male lymphoblasts, Xi and Xa mouse-human hybrids and an HT1080 (male fibrosarcoma) cell line containing a DOX-inducible *XIST* transgene (HT1080*XISTi*). Sensitivity of biological triplicates ± standard deviation is shown relative to an unsensitive region. *JPX* is used as a positive control. *p = 0.05-0.01, **p = 0.01-0.001, ***p < 0.001, n.s. = not significant.

To locate candidate *cis*-regulatory elements for *XIST* we surveyed the *XIC* for DHS sites identified by genome-wide DHS-seq studies. Interestingly, the strongest DHS sites within the *XIC* are located within the *XIST* CpG island, ~1.4 kb downstream of the *XIST* promoter, and are female-specific. We carried out a DNase I hypersensitivity assay at two regions, DHS 200b.1, which lies within the CpG island, and DHS 200a.1 which lies 518 bp downstream of the CpG island (Figure 1A). DHS 200b.1 was hypersensitive to digestion by increasing concentrations of DNase I in female lymphoblast cells but not in male lymphoblast cells, while DHS 200a.1 was not hypersensitive in female or male lymphoblast cells (Figure 1B). The difference between male and female sensitivity at DHS 200b.1 hinted at an Xi to Xa difference so we used Xi and Xa-containing mouse-human hybrid cells to assess the chromatin of the Xa and Xi separately. We saw a significant increase in sensitivity at DHS 200b.1 in an Xi-containing mouse-human hybrid cell line and modest sensitivity at DHS 200a.1, while no effect of DNase treatment was seen at either region in an Xa hybrid cell line (Figure 1B) which demonstrated that the DHS site was specific to the Xi. The difference in sensitivity at region DHS 200a.1 between female lymphoblast cells and Xi-containing mouse-human hybrid cells is likely a reflection of the Xa in female cells masking weak sensitivity in the region. Interestingly, HT-1080 male fibrosarcoma cells transfected with a DOX inducible *XIST* cDNA clone (HT1080*XISTi*) [37] showed no hypersensitivity before or after DOX induction of the full-length XIST transcript at either DHS 200b.1 or DHS 200a.1 (Figure 1C). This may mean that establishment of the hypersensitivity within the CpG island of *XIST* occurs developmentally and is not recapitulated in differentiated somatic cells.

### 2.2 Promoter Activity of DHS 200b.1

**Figure 2:**
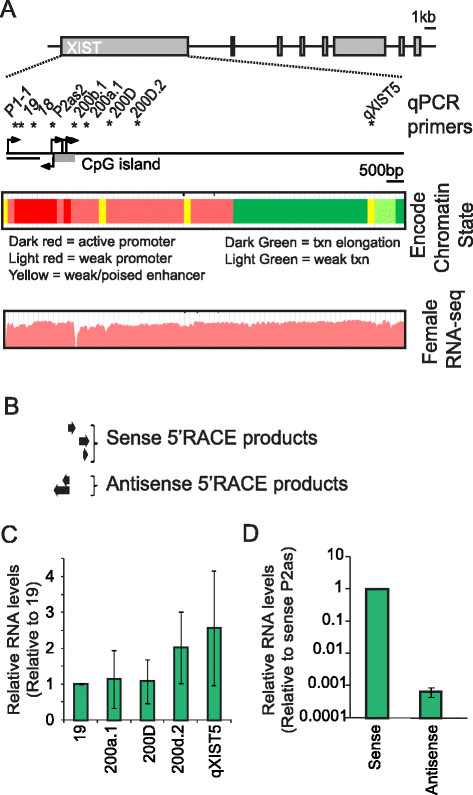
**Xi-specific DHS site correlates with an actively transcribing P2 promoter. A)** Schematic of *XIST* exon 1 indicating the locations of primers used for qRT-PCR analysis. The outlined boxes are UCSC tracks showing chromatin state segmentation as determined by compiled ChIP-seq data (upper) and RNA-seq data for the region (lower) [38],[39]. **B)** Sequenced 5‘RACE products with arrows indicating transcription orientation. **C)** Levels of XIST upstream and downstream of P2 determined by qRT-PCR (shown as biological triplicates ± standard deviation). **D)** Strand-specific qRT-PCR of P2as transcripts relative to sense XIST (average of biological triplicates ± standard deviation).

Genome-wide ChIP-seq data on the UCSC genome browser shows peaks for several promoter-associated proteins and chromatin modifications in female cell lines overlapping the *XIST* CpG island and DHS 200b.1. These marks include RNA Polymerase II, H3K4me3, H3K27ac, H3K9ac [38] and over a dozen transcription factors including YY1, a protein implicated in regulation of XCI in mouse [21]. Moreover, chromatin state segmentation studies identify the DHS 200b.1 region as an active promoter [39] (Figure 2A) and studies in mouse have suggested the presence of an alternative promoter approximately 1.5 kb within the *Xist* gene [10]. To address the question of promoter activity for DHS 200b.1 we performed 5′ Rapid Amplification of cDNA Ends (RACE) assays on a region overlapping the hypersensitive site and the CpG island of *XIST* (Figure 2A)*.* 5′ RACE uncovered three transcription start sites (TSS) in the sense orientation spanning a region of 404 bp across the CpG island (black arrows in Figure 2A). We therefore conclude that there is an alternative promoter of human *XIST* that we designate as P2. In the antisense orientation a TSS located at the 3′ edge of the CpG island was also found, which we designate P2as (Figure 2B). Using qPCR primers across XIST exon 1 no significant increase in transcription was found downstream of P2, which is corroborated by RNA-seq data in female cells [38], suggesting that P2 is unlikely to be the dominant *XIST* promoter (Figure 2C). Similarly, using strand-specific qRT-PCR we found P2as transcripts to be at levels 0.001% of sense *XIST* transcripts, reducing the likelihood of these having a regulatory function (Figure 2D). Since XCI and the bulk of *XIST* regulation must occur early in human development we also examined the male human ES cell line CA1S for P2 or P2as function, which allows for examination of negative regulators of *XIST* and avoids the variable XCI patterns in female ES cells. We found transcription throughout the whole gene body of *XIST* and between 8683 bp to 9934 bp beyond the 3′ end of *XIST* (Additional file 1: Figure S1A,B). Strand-specific RT-PCR assays determined that transcription is in antisense orientation, providing the first evidence for antisense transcription reaching the *XIST* promoter in humans (Additional file 1: Figure S1C). The faint RT-PCR bands, however, suggested low-level transcription, which was verified by qRT-PCR, indicating that the antisense transcript in this region is at 0.00012% of *XIST* expression levels in a somatic female lymphoblast. The region between as well as 5′ of these two primers contains an enrichment of endogenous retrovirus in the antisense orientation and an Alu element so it is possible that transcription is initiating from an endogenous retroviral long terminal repeat promoter.

### 2.3 YY1 binding at the P2 region

**Figure 3:**
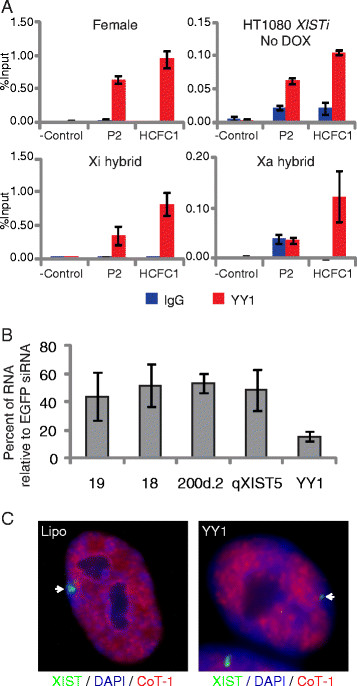
**YY1 binding to region of P2 promoter is Xi-specific and upregulates P1 transcription. A)** Chromatin immunoprecipitation (ChIP) of YY1 binding to P2 shown relative to input, at P2, a negative control region within *XIST*, and a positive control region (*HCFC1* gene promoter) for female (IMR-90), HT1080*XISTi* transgenic *XIST*, and Xi and Xa-containing hybrids. A representative ChIP is shown ± standard deviation of q-PCR triplicates; with replicate experiments showing the same pattern but variable levels of IP relative to input. **B)** Q-PCR following 72 h of siRNA mediated knockdown of YY1 in IMR-90 female cells demonstrates a decrease in expression of XIST, both upstream (19, 18) and downstream of P2 (200d.2, qXIST5) relative to cells with a control (EGFP) knockdown. Levels of YY1 dropped to less than 20% confirming successful knockdown. **C)** FISH for XIST RNA following YY1 knockdown. Left panel shows representative IMR-90 female cells treated with lipofectamine alone (Lipo) and right panel shows cells after YY1 knockdown. Arrows indicate location of XIST RNA signal (green) which was substantially reduced after YY1 knockdown.

We next asked whether P2 activity and hypersensitivity at DHS 200b.1 was correlated with binding of YY1 *in vivo* using chromatin immunoprecipitation (ChIP). YY1 was found to be enriched at P2 in both female cells and Xi-containing hybrid cells but not Xa-containing hybrid cells, indicating Xi-specific binding (Figure 3A). We also found YY1 binding at P2 in inducible XIST HT1080*XISTi* cells before induction by DOX, despite these cells lacking a DHS, demonstrating that YY1 binding is insufficient to establish hypersensitivity in the P2 region. Since YY1 binding appeared to be independent of the DHS associated with P2 we wished to address the impact of down-regulating YY1 on the transcription of *XIST*. After 72 h of siRNA-mediated knockdown of YY1 in IMR90 female cells we saw a 50% decrease in expression of *XIST*, both upstream and downstream of P2, suggesting that YY1 may be a regulator of transcription at P1 (Figure 3B). By fluorescent *in situ* hybridization for the XIST RNA we observed only a small focus of expression in 41/86 (48%) of cells and no detectable signal in 35/86 (40%) of cells, with substantial delocalization of the RNA in the remaining 12% of cells (Figure 3C).

### 2.4 DNA Methylation at P2 CpG island

**Figure 4:**
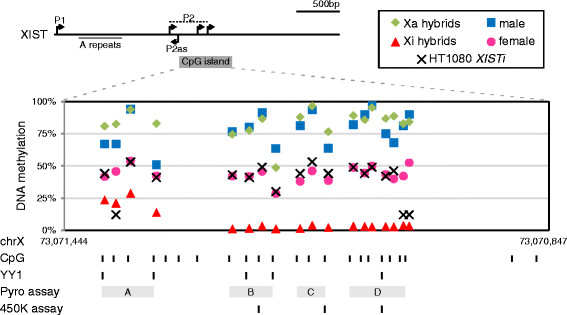
**DNA methylation of the CpG island containing XIST P2.** Schematic of the 5′ end of *XIST* showing P1 and P2 with the intervening 5′ A repeats. Methylation levels shown for each site are the average of two male cell lines, three female cell lines, two Xa and four Xi-containing hybrids, and the HT1080 with *XIST* transgenic line. Shown below is the location of the pyrosequencing assays **(A to D)** designed to assess methylation at 18 of the 23 CpG sites in the intermediate density CpG island. Also shown are the YY1 sites (based on consensus sequence 5′-CCGCCATNTT-3′ from http://www.uniprot.org/uniprot/P25490) as well as the CpGs analysed by Illumina 450 K Bead Chip.

As diagrammed in Figure 2, the region around P2 is classified as a CpG island according to the more relaxed ‘intermediate’ classification that requires GC content greater than 50% and an observed CpG frequency to expected CpG frequency of greater than 0.48 over at least 200 bp [40]. The region 5′ to the CpG island has been previously reported to be differentially methylated by restriction enzyme analysis [12] and both the region immediately upstream of the *XIST* major promoter (P1 – two sites) and three sites within the P2 island (see Figure 4) are present on the Illumina 450 K bead chip and show intermediate methylation in females and hypermethylation in males [41]. To more completely assess the methylation across this region, including across the differentially bound YY1 sites, we designed four pyrosequencing assays to analyse 18/21 CpG dinucleotides in the CpG island as shown in Figure 4. In general, male samples or Xa-containing hybrids showed over 60% methylation, while the Xi-containing hybrid had low methylation (see also Table 1). Females, and also the HT1080*XISTi* male cell line with a single copy *XIST* transgene, showed intermediate methylation.

**Table 1:** Activities observed at XIST P2 region

	**DHS**^**@**^	^**+**^**P2 activity**	^**++**^**YY1 binding**	^**+++**^**DNA methylation**
**Male**	-	-	-	78%
**Female**	+	+	+	44%
**Xa Hybrid**	-	-^#^	-	83%
**Xi Hybrid**	+	+*	+	7%
**HT1080** ***XISTi***	-	-	+	39%
**Male hES**	-**	-	-**	80%**

^@^DHS: DNase Hypersensitive Site (from Figure 1; except for male hES (**) from UCSC).

^+^P2 activity from RACE in Figure 2 or ^#^[2]. *band observed with RACE, but not sequence-confirmed.

^++^YY1 binding from Figure 3, except ** from UCSC.

^+++^DNA methylation is an average from 18 sites as shown in Figure 4.

## 3 Discussion

DHS sites serve as a molecular mark to identify important regulatory elements, and a strong DHS within the human *XIST* gene has been shown to be female-specific [42], suggesting an important regulatory role in X inactivation. Human ES cells have not provided as tractable a model for the study of human *XIST* regulation as mouse ES cells have been for the study of the early events of XCI in mouse. Therefore, we have assessed the role of the regulatory region demarcated by the DHS site through correlation of features in a variety of model cell types, as summarized in Table 1. The female-specificity of the DHS can be attributed to the presence of an Xi, as shown by the analysis of the somatic cell hybrids. The chromatin marks for this region suggest the presence of promoter activity, and we verified that ′P2′ is the origin for a variety of transcripts. Presence of the DHS was completely concordant with P2 promoter activity; however, in mouse, the P2 region has also been shown to be the site of binding of YY1. Therefore we assessed YY1 binding by ChIP and confirm that YY1 binds to the human P2 region. YY1 binds the transgenic *XIST*, which was integrated in somatic cells, and therefore YY1 binding does not require passage of the DNA through development. Furthermore, in these HT1080-derived transgenic cells, no evidence for P2 transcriptional activity is observed ([37], data not shown), separating P2 function from YY1 binding. Therefore, P2 function is completely concordant with the DHS presence, and may require additional factors, that are perhaps acquired developmentally, for establishment. While the DHS within the *XIST* island is the only female-specific site near the *XIC* regulatory region, two other female-specific DHS sites were reported on the human X chromosome, and all were associated with long non-coding RNA loci [42]. The other two sites, *FIRRE* (LOC286467; [43]) and the noncoding RNA LOC550643 are also affiliated with complex tandem repeats, differential methylation and CTCF binding, reminiscent of the DXZ4 region [44].

Both humans and mice demonstrate a P2 within the body of *XIST/Xist*, and in both cases, transcription from this promoter would generate a transcript lacking the 5′A repeats that are critical for the silencing function of XIST/Xist [37],[45], raising the question of whether P2 transcription might be secondary to another function for the region. The first alternative role we considered was the generation of other transcripts. All transcription in the region was only observed from the active *XIST/Xist* gene on the Xi, unlike some short RNAs that are transcribed from repressed polycomb target genes or imprinted loci [46]. In mice, there is a 1.6 kb transcript, called RepA, that is expressed from the active locus, and thus the future Xi; however, as the name suggests, this transcript contains the A repeats [47]. We did identify an antisense transcript; however, the very low abundance suggests that this transcript is also not functional; although higher levels of such an antisense might interfere with P1 transcription, and therefore the sense P2 transcript may be involved in preventing P2 antisense transcription from silencing functional P1 transcription. Interestingly, we also observe an antisense transcript across XIST in human ES cells; however, again, this transcript is at such low levels it seems unlikely to reflect a biologically relevant function.

If generation of biologically active transcripts is not the principal role of this region, transcription in the region might be an indirect outcome of YY1 binding to tether the RNA as YY1 binding has recently been shown to act as a ‘tether’ for the Xist RNA to bind to the DNA of the chromosome. YY1 binding in the region is seen to be female-specific in browser tracks, and we confirmed that this YY1 binding is Xi-specific as it is observed in Xi, but not Xa-containing hybrids. Interestingly, in these somatic cell hybrids, despite binding of YY1, the RNA is not tethered to the chromosome, and drifts away [48], implicating further species-specific factors in the ability of DNA to interact with the *cis*-transcribed RNA. We also observed YY1 binding in HT1080*XISTi* transgenic cells; however, these cells fail to demonstrate P2 activity, suggesting that P2 transcription is not simply a function of YY1 binding. To determine if YY1 binding impacted localization or transcription of *XIST* we knocked down YY1 with siRNA. Interestingly, we did observe a reduction in XIST RNA abundance, across the gene, upon knock-down of YY1. This could reflect a role for YY1 in transcription from P1**.** An alternative explanation could be that delocalized XIST is more rapidly degraded; however, in the mouse/human somatic cell hybrids the delocalized XIST still had a half-life equivalent to localized XIST [48]. There could also be downstream effects due to the knockdown of the important YY1 protein that have an indirect effect on *XIST* transcription; however, reporter assays have shown that inclusion of this region augments transcription [11], supporting an enhancer action for this region.

The binding of YY1 was seen to be concordant with lack of methylation of the CpG island within *XIST*. This region is normally differentially methylated in females, and differentially methylated regions (DMRs) are often associated with mono-allelic expression, similar to that observed for *XIST*, and also imprinted genes. Such DMRs often overlap binding regions for the insulator/enhancer blocking factor CTCF [49], and there is CTCF binding downstream of the *XIST* DMR and upstream of P1; however, neither is sex-specific [41] suggesting that they are not primary regulators of *XIST* expression. While binding of YY1 to the imprinted *Peg3* gene was suggested to be methylation-sensitive [50], the binding of YY1 to DXZ4 is similarly restricted to the hypomethylated allele, however binding *in vitro* is not blocked by DNA methylation [51]. Thus, while YY1 binding is concordant with hypomethylation, it is not clear wehther DNA methylation prevents YY1 binding to the Xa. Genome-wide analyses have shown many additional transcription factors binding the P2 DMR region, some combination of which likely contributes to activation of P2 transcription and establishment of the DHS, possibly during early development.

## 4 Conclusions

We report an Xi-specific DHS site within the human *XIST* locus. This site overlaps a weak CpG island that shows differential methylation between the Xa and Xi. This methylation spans five consensus YY1 binding sites, and, consistent with ENCODE data, YY1 binds in a female-specific manner. The DHS is concordant with P2 promoter activity; however, transcription from the conserved P2 results in a transcript of unknown function that lacks the A repeats previously shown to be critical for silencing. In addition to P2 activity, this region apparently functions as both an enhancer and RNA tether, as knockdown of YY1 reduces P1 transcription and also results in loss of XIST localization to the Xi.

## 5 Methods

### 5.1 DNase I hypersensitivity

2,000,000 cells were harvested and lysed using 0.1% NP40 in resuspension buffer (RSB) (10 mM Tris pH 7.4, 10 mM NaCl, 3 mM MgCl2). Nuclei were resuspended in RSB and digested with 0U, 10U, 20U or 40U of DNase I for 10 min at 37°C. Digestion was stopped and DNA was extracted using 0.8 mL of DNAzol (Invitrogen) followed by ethanol precipitation. The DNA was diluted to a final concentration of 20 ng/μl, to be used in qPCR. Primers for qPCR were designed to span the test hypersensitive site (200b.1 and 200a.1) as well as a positive control region (JPX) and an insensitive region (XIST3′5′). Hypersensitivity was calculated by normalizing each DNase I concentration to the insensitive region and then results from each DNase I concentration were plotted as a fold difference from the untreated sample.

### 5.2 Tissue culture and cell lines

Mouse-human somatic cell hybrid cell lines, t75-2maz 34-1a (containing a human Xi) and t60-12 (containing a human Xa) [52] were cultured at 37°C in alpha Minimum Essential Medium (MEM) supplemented with 7.5% fetal calf serum (PAA Laboratories Inc), 1% penicillin/streptomycin (Life Technologies) and 1% L-glutamine (Life Technologies). The GM11200 and GM7057 male lymphoblast cell lines and GM11201, and GM7350 female lymphoblast cells lines (Coriell cell repository) were maintained in Roswell Park Memorial Institute (RPMI) medium supplemented with 15% fetal calf serum (PAA Laboratories Inc), 1% penicillin/streptomycin (Life Technologies) and 1% L-glutamine (Life Technologies). The IMR90 female fibroblast cell line was cultured with 10% fetal calf serum (PAA Laboratories Inc) and 1% L-glutamine (Life Technologies). The HT1080*XISTi* transgenic cell line containing a DOX-inducible *XIST* was cultured in Dulbecco’s Modified Eagle Medium (DMEM) supplemented with 10% fetal calf serum (PAA Laboratories Inc), 1% penicillin/streptomycin (Life Technologies) and 1% L-glutamine (Life Technologies). CA1S cells were cultured as described [53] on Matrigel (BD Biosciences) coated 6-well plates in mTeSR1 basal medium (STEMCELL) supplemented with mTeSR1 5x supplement (STEMCELL) and passaged using Accutase (STEMCELL). All lines were cultured at 37°C.

### 5.3 PCR and quantitative PCR

PCR was performed with 100 ng of genomic DNA template, 1U Taq polymerase, 0.2 mM dNTPs, 1.5 mM MgCl2, 1X PCR buffer (all from Invitrogen) and 0.5 μM of both forward and reverse primers. PCR was performed using 30–40 cycles of [95°C for 30 s, 55°-60°C for 30 s, 72°C for 1 min]. Quantitative PCR (qPCR) was performed using the StepOnePlusTM Real-Time PCR System (Applied Biosystems) and the qPCR reaction mix was composed of 0.2 mM dNTP mix, 2.5 mM MgCl2, 1X HS reaction buffer, 1X EvaGreen dye (Biotum), 0.25 μM forward and reverse primer, and 0.8 U Maxima Hot Start Taq (Fermentas) or AptaTaq Fast DNA polymerase (Roche) and cycling conditions were as follows: 95°C for 5 min, followed by 40 cycles of [95°C for 15 s, 60°C for 30 s, 72°C for 1 min]. Each sample and negative control was assayed in triplicate.

### 5.4 RNA extraction and reverse transcription

RNA was extracted using Trizol (Invitrogen) following the manufacturer’s instructions. RNA was treated using the DNA-free kit (Ambion) to remove genomic DNA contamination according to the manufacturer’s instructions and RNA concentrations were determined using a spectrophotometer. Reverse transcription (RT) of RNA was carried out using 2 μg of RNA, 1x first strand buffer (Invitrogen), 0.01 mM Dithiothreitol (DTT) (Invitrogen), 0.125 mM dNTPs, 1 μL random hexamers, 1 μl RNase Inhibitor (Fermentas) and 1 μL (1U) of Moloney Murine Leukemia Virus reverse transcriptase (M-MLV) and water was added to a total volume of 20 μL. Reactions without RT were also carried to out to test for complete removal of genomic DNA contamination. Reaction mixes were incubated for 1 h at 42°C and heat inactivated by incubating at 95°C for 5 min.

For strand-specific RT reactions 2 μg of RNA was mixed with 0.50 μM dNTPs and 2 pmol of sense or antisense gene specific primer with a T7 sequence tag on the 5′ end of the primer and then heated to 70°C for 5 min. Following this incubation the tubes were placed on ice for 1 min and then mixed with 1x first strand buffer (Invitrogen), 0.005 DTT, 1 μL (1U) RNase Inhibitor (Fermentas) and 1 μL (1U) Superscript III. Reactions minus RT were also carried to out to test for complete removal of genomic DNA contamination. Reaction mixes were incubated for 1 h at 55°C and heat inactivated by incubating at 95°C for 5 min.

### 5.5 5′ and 3′ rapid amplification of cDNA ends

5′ RACE was performed using the First Choice RLM-RACE kit (Ambion) as per the manufacturer’s instructions. 5′ RACE cDNA was amplified using nested PCR reactions (components as above) with 35 cycles each and annealing temperatures of 57°C, for the outer PCR reaction, and 59°C for the inner PCR reaction. 3′ RACE was performed using the First Choice RLM-RACE kit (Ambion) as per the manufacturer’s instructions. PCR products from both 5′ and 3′ RACE were analyzed using 2% gel electrophoresis and PCR products purified with the QIAquickGel extraction kit (Qiagen) for Sanger sequencing.

### 5.6 siRNA-mediated knockdown

Knockdown was performed according to the manufacturer’s instructions. Briefly, 100,000 cells were seeded into each well of a 24 well tissue culture plate. After 24 hrs the cells were transfected with 2 μL of Dharmafect 4 transfection reagent (Thermo Scientific) and 0.05 μM of siGenome SMARTpool siRNA (M-011796-02 for YY1) and harvested after a further 72 hrs.

### 5.7 Chromatin immunoprecipitation

Briefly, 5,000,000 cells were crosslinked using with 1% formaldehyde and lysed with cell lysis buffer (10 mM Tris–HCl, pH 8, 10 mM NaCl, 3 mM MgCl2, 0.5% Igepal, protease inhibitor). Nuclei were resuspended in nuclear lysis buffer (1% SDS, 5 mM EDTA, 50 mM Tris–HCl, pH 8, protease inhibitor) and sonicated for 10 min to a chromatin range of 150 bp to 500 bp. NaCl was added to the chromatin to a final concentration of 150 mM and then diluted in ChIP dilution buffer (0.01% SDS, 1.1% Triton X-100, 150 mM NaCl, 16.7 mM Tris–HCl pH 8). 1% chromatin was collected for input DNA and remaining chromatin was incubated with either 10ug IgG antibody (I8140; Sigma) or 10 ug YY1 antibody (sc-1703; Santa Cruz Biotechnology, Inc.) overnight. Chromatin and antibody was incubated with blocked protein A:G beads and then washed with low-salt buffer (0.1% SDS, 1% Triton X-100, 2 mM EDTA, 20 mM EDTA, 20 mM Tris–HCl, pH 8, 150 mM NaCl), high salt buffer (0.1% SDS, 1%Triton X-100, 2 mM EDTA, 20 mM Tris–HCl, pH 8, 500 mM NaCl), LiCL buffer (0.25 M LiCl, 1% Igepal, 1% Deoxycholate, 1 mM EDTA, 10 mM Tris–HCl, pH 8) and TE buffer (10 mM EDTA, 10 mM Tris–HCl, ph 8) and then eluted with elution buffer (1% SDS, 0.1 M NaHC3). Precipitated chromatin and input DNA were was uncrosslinked overnight in elution buffer and 0.192 M NaCl and proteinase K and purified using QIAquick DNA purification columns. Locus-specific enrichment was quantified using qPCR.

### 5.8 Statistical analysis

Statistical analysis was performed using GraphPad Prism 5.02. One-way ANOVA was used to test for significance in DHS experiments between different concentrations of DNase I.

### 5.9 FISH analysis

RNA FISH was carried out as described by [54]. An *XIST* probe and human Cot-1 DNA (Invitrogen) probe were fluorescently labelled by nick translation (Abbott Molecular Inc.) using SpectrumGreen-UTP (Vysis) for *XIST* and SpectrumRed-UTP (Vysis) for Cot-1. The *XIST* probe used, HbC1a, covers a ~1.6 kb region of *XIST* including most of the repeat A region. Briefly, cells grown on glass coverslips were permeabilized in 0.5% Triton X-100 for eight minutes and then fixed in 4% paraformaldehyde (Electron Microscopy Sciences) for eight minutes. Cells were hybridized overnight with 150 ng XIST probe and 150 ng human Cot-1 DNA probe. Following a series of post-hybridization rinses, the coverslips were counterstained with DAPI and mounted on glass slides with Vectashield (Vector Laboratories) for imaging.

### 5.10 DNA methylation analysis by pyrosequencing

Three female cell lines (GM08399, GM05396 and GM08134), two male cell lines (GM7057 and GM1200), two Xa hybrids (t60-12 and AHA-11aB1) and four Xi hybrids (t86-B1maz-1b, t75-, tHM-1A and tHM-34-2A) were each assessed for methylation using four pyrosequencing assays. Pyrosequencing was performed using a Pyromark ID machine as previously outlined in Cotton *et al.*[55]. Briefly, approximately 25 ng of bisulfite converted DNA was PCRed along with 1X PCR Buffer (Qiagen), 0.2 mM dNTPs, 0.025 U HotStart Taq DNA Polymerase (Qiagen), 0.25 mM forward primer and 0.25 mM reverse primer (listed in Additional file 2: Table S1). PCR cycling conditions were the same for all four Pyrosequencing assays, 95°C for 15 min, 50 cycles of 94°C for 30 s, 55°C for 30 s, 72°C for 60 s, followed by a final step of 72°C for 10 min.

## Abbreviations

XCI: X-chromosome inactivation

Xi: Inactive X chromosome

XIC: X-inactivation center

FISH: Fluorescent *in situ* hybridization

## Competing interests

The authors declare that they have no competing interests.

## Authors’ contributions

AGC designed and carried out the studies described and drafted the manuscript. CJB participated in the study design, results interpretation and manuscript writing. AMC designed and analysed the methylation assays and the preparation of the figures, ADK performed the FISH analysis. All authors have read and approved the final manuscript.

## Additional files

## Supplementary Material

Additional file 1: Figure S1 Transcription at the XIST locus in CA1S male hES cells **A)** Schematic of XIST indicating primer positions (*) used in RT-PCR. **B)** RT-PCR in CA1S cells at the XIST locus. **C)** Strand specific RT-PCR to determine orientation of transcription. Click here for file

Additional file 2: Table S1 Primers used in this manuscript. Click here for file
