# Supplementary figures and images for: Differentially methylated CpG island within human XIST mediates alternative P2 transcription and YY1 binding
Source: BMC Genet. 2014 Sep 9;15:89. doi: 10.1186/s12863-014-0089-4 (PMC4363909; doi:10.1186/s12863-014-0089-4)

A

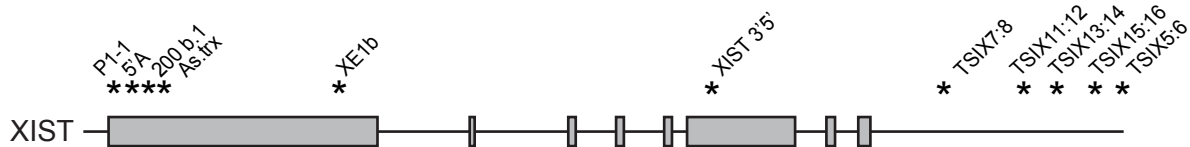

B

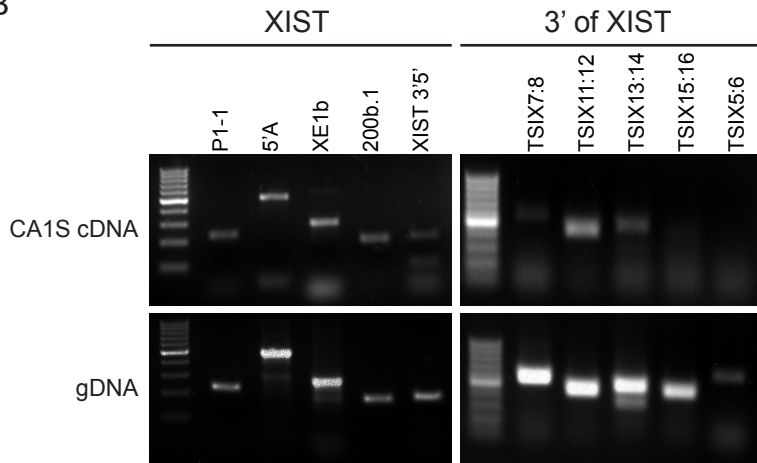

C

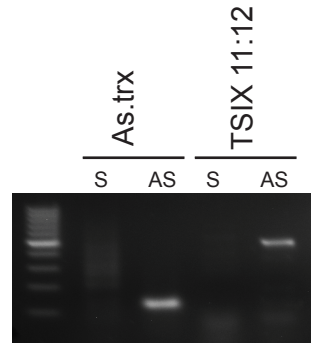

Supplement: Additional file 1: Figure S1 — Transcription at the XIST locus in CA1S male hES cells A) Schematic of XIST indicating primer positions (*) used in RT-PCR. B) RT-PCR in CA1S cells at the XIST locus. C) Strand specific RT-PCR to determine orientation of transcription. [file s12863-014-0089-4-S1.pdf]
