# Supplementary material for: Differentially methylated CpG island within human XIST mediates alternative P2 transcription and YY1 binding
Source: BMC Genet. 2014 Sep 9;15:89. doi: 10.1186/s12863-014-0089-4 (PMC4363909; doi:10.1186/s12863-014-0089-4)
Supplement: Additional file 2: Table S1 — Primers used in this manuscript. [file s12863-014-0089-4-S2.docx]

**Additional file 2: Table S1: List of Primers (Shown 5’ to 3’)**

| **Purpose** | **Name** | **Sequence** |
| --- | --- | --- |
| RT-PCR and qPCR | 200b.1F | TGTCCATCCCACCTTTTCTC |
|  | 200b.1R | TCTTTGCTGTGTGCTTTTCG |
|  | 200a.1F | ATCCGACCCCAGCATTAGC |
|  | 200a.1R | GCTCCAGGCCTGCTTGGT |
|  | JPX F | GCGGAGGCATTTAGGTAGTG |
|  | JPX R | GGCGAGTTTCTGGACTTTTG |
|  | XIST 5’ | GAAGTCTCAAGGCTTGAGTTAGAAG |
|  | XIST 3’ | TTGGGTCCTCTATCCATCTAGGTAG |
|  | P1-1F | TGTCAACCAAAAATGATTCCA |
|  | P1-1R | TCTCTGCACTTGGGGTTCTT |
|  | 19F | AACTGATCCACAAAAAACAGAGATGT |
|  | 19R | TCTTCTTGACACGTCCTCCATATTT |
|  | 200DF | AGAGGACACCAGACCACAGC |
|  | 200DR | TGTGCTGGTCATTTTCTTTGA |
|  | 200D.2F | GGATTCTCCAGAAGCACAGC |
|  | 200D.2R | AGCACTCTGAACCCCATTTG |
|  | qXIST5F | CCTAGTTCAGGCCTGCTTTTCAT |
|  | qXIST5R | TCAGCCCATCAGTCCAAGATC |
|  | qWithin.XIST.DHS.1F (P2) | ACAAAGCCTCGCCCATCAT |
|  | qWithin.XIST.DHS.1R (P2) | CCAGTTCTGTCGCAGTGTTCA |
|  | HCFC1.F | TTCCACTGCACACCAAACTC |
|  | HCFC1.R | CGGGGCCTAACTCTAGCTCT |
|  | 18F | TGCACTCTCTGGAATATCTACACTTTT |
|  | 18R | ATCTGAACACGCCCTTAGCTTAA |
|  | YY1F | ACCTGGCATTGACCTCTCAGA |
|  | YY1R | TTTTTCTTGGCTTCATTCTAGCAA |
|  | 5’AF | TGACTTCCTCTGCCTGACC |
|  | 5’AR | GATTCCCTTCCCCTCTGAAC |
|  | XE1BF | AGTGCCAAATGCCAGGATAC |
|  | XE1BR | AATGCTGGTAAAGCCCACAC |
|  | TSIX7 | CAGTACCAGCATTCTCAGTG |
|  | TSIX8 | CCACTCTCATTGTCATTGCG |
|  | TSIX11 | CCAGCTGCAACTCAGATGTA |
|  | TSIX12 | CCTTCTTCTCAGAGACTCCT |
|  | TSIX13 | CTGATAAGTGACCAGTCACC |
|  | TSIX14 | TGAAGACACTGGCCTTGACA |
|  | TSIX15 | TGGCACACGTATGTGGTTCT |
|  | TSIX16 | CTCTGAGTCTTCCTATGACC |
|  | TSIX5 | TTGGGGATGGAGAATAGGTG |
|  | TSIX6 | CCTGATCTGAGTTATGGCAC |
| Strand – specific RT- PCR | t7.P2as-2F | **TAATACGACTCACTATAGGGAGA**CTGCCTCCCGATACAACAAT |
|  | t7.P2as-2R | **TAATACGACTCACTATAGGGAGA**GATAGCAGGTCAGGCAGAGG |
|  | t7.AS.trx.withinXISTF | **TAATACGACTCACTATAGGGAGA**ACAGAGGAATGGAGGGAGGT |
|  | t7.AS.trx.withinXISTR | **TAATACGACTCACTATAGGGAGA**AGGGGCCACGTGTATGTCT |
|  | TSIX11.t7 | **TAATACGACTCACTATAGGGAGA**CCAGCTGCAACTCAGATGTA |
|  | TSIX12.t7 | **TAATACGACTCACTATAGGGAGA**CCTTCTTCTCAGAGACTCCT |
| 5’RACE | P2a outer | ATCTGAACACGCCCTTAGCTTAA |
|  | P2a inner | TGACTTCCTCTGCCTGACCT |
|  | P2b outer | AACACTGCGACAGAACTGGA |
|  | P2b inner | CTGCCTCCCGATACAACAAT |
|  | P2c outer  | CAATTCCACCCCCATTTCTA |
|  | P2c inner | TGTCCATCCCACCTTTTCTC |
|  | P2d outer | AGAGGACACCAGACCACAGC |
|  | P2d inner | ATCCGACCCCAGCATTAGC |
|  | 5’ RACE outer | GCTGATGGCGATGAATGAACACTG |
|  | 5’ RACE inner | CGCGGATCCGAACACTGCGTTTGCTGGCTTTGATG |
| Pyrosequencing | XIST_A - F | TGGAAGGTTAGGAAAGATTAAGGTGTAG |
|  | XIST_A - R | BIOTIN - TGTTAAGTGGTTAGTATGGTGGTGG |
|  | XIST_A - S | TTAGGAAAGATTAAGGTGTA |
|  | XIST_B - F | GGTTAGTATGGTGGTGGATATGT |
|  | XIST_B - R | BIOTIN - GGATAGTGTTGGATTGTTGTATAATTT |
|  | XIST_B - S | GGTGGTGGATATGTG |
|  | XIST_C - F | GGTTAGTATGGTGGTGGATATGT |
|  | XIST_C - R | BIOTIN - GGATAGTGTTGGATTGTTGTATAATTT |
|  | XIST_C - S | TGTTTTTGTTGTGTGTTT |
|  | XIST_D - F | GGTTAGTATGGTGGTGGATATGT |
|  | XIST_D - R | BIOTIN - GGATAGTGTTGGATTGTTGTATAATTT |
|  | XIST_D - S | GTTGTTATATGTATATTATGGTTTT |
